# Predictors of prolonged length of stay in adult patients with respiratory syncytial virus infections – a multi-center historical cohort study

**DOI:** 10.3389/fmicb.2024.1385439

**Published:** 2024-04-04

**Authors:** Ambreen Malik, Susan Szpunar, Mamta Sharma, Leonard B. Johnson, Louis Saravolatz, Ashish Bhargava

**Affiliations:** ^1^Division of Infectious Diseases, Department of Internal Medicine, Ascension St. John Hospital, Detroit, MI, United States; ^2^Department of Biomedical Investigations and Research, Ascension St. John Hospital, Detroit, MI, United States; ^3^Thomas Mackey Center for Infectious Disease Research, Ascension St. John Hospital, Detroit, MI, United States

**Keywords:** respiratory syncytial virus (RSV), predictors, risk factors, hospital length of stay, prolonged hospital length of stay

## Abstract

**Objectives:**

Several studies have reported risk factors for severe disease and mortality in hospitalized adults with RSV infections. There is limited information available regarding the factors that affect the duration of a patient’s hospital length of stay (LOS).

**Methods:**

This was a multicenter historical cohort study of adult patients hospitalized for laboratory-confirmed RSV in Southeast Michigan between January 2017 and December 2021. Hospitalized patients were identified using the International Classification of Diseases, Tenth Revision 10 codes for RSV infection. Mean LOS was computed; prolonged LOS was defined as greater than the mean.

**Results:**

We included 360 patients with a mean age (SD) of 69.9 ± 14.7 years, 63.6% (229) were female and 63.3% (228) of white race. The mean hospital LOS was 7.1 ± 5.4 days. Factors associated with prolonged LOS in univariable analysis were old age, body mass index (BMI), smoking status, Charlson Weighted Index of Comorbidity (CWIC), home oxygen, abnormal chest x-ray (CXR), presence of sepsis, use of oxygen, and antibiotics at the time of presentation. Predictors for prolonged LOS on admission in multivariable analysis were age on admission (*p* < 0.001), smoking status (*p* = 0.001), CWIC (*p* = 0.038) and abnormal CXR (*p* = 0.043).

**Interpretation:**

Our study found that age on admission, smoking history, higher CWIC and abnormal CXR on admission were significantly associated with prolonged LOS among adult patients hospitalized with RSV infection. These findings highlight the significance of promptly recognizing and implementing early interventions to mitigate the duration of hospitalization for adult patients suffering from RSV infection.

## Introduction

Respiratory Syncytial Virus (RSV) causes significant morbidity and mortality, resulting in 60,000 to 160,000 hospitalizations and 6,000 to 10,000 fatalities annually among adults aged 65 years or older (Centers for Disease Control and Prevention, n.d.). In recent years, it has become increasingly recognized that RSV can trigger exacerbations of chronic obstructive pulmonary disease (COPD), asthma, and congestive heart failure (CHF) in older adults (Centers for Disease Control and Prevention, n.d.; Ackerson et al., 2019; Branche et al., 2022; Sah et al., 2023). Underlying medical conditions may significantly influence the severity of RSV-related disease (Walsh et al., 2013; Chatzis et al., 2018). Hospitalization of these high-risk individuals causes a substantial economic impact on the healthcare system, with an annual US cost to be estimated at around $1.2 billion (Choi et al., 2022). The length of time a patient stays in a hospital is called “length of stay” (LOS). This duration is an indirect indicator of the patient’s sickness level and is directly related to the financial costs of hospitalization. As there is a focus on providing high-value medical care and improving healthcare quality, a hospital’s average length of stay is considered a measure of the quality of healthcare delivery (Englert et al., 2001).

Previous studies have reported the factors that can predict the severity of RSV and the likelihood of complications among hospitalized adult patients (Chatzis et al., 2018; Kieke et al., 2020; Atamna et al., 2021; Hartnett et al., 2022; Njue et al., 2023). Similarly, studies have identified the factors that indicate poor survival among hospitalized adults infected with RSV (Lee et al., 2013). A recent MMWR report showed that patients hospitalized adults aged ≥60 years with RSV were more likely to die compared to patients hospitalized with influenza affecting their hospital LOS (Surie et al., 2023). The incidence of complications, including pneumonia, bacterial co-infections and the need for intensive care admission, had been higher among patients hospitalized with RSV than in those with influenza, which can prolong the hospital LOS (Lee et al., 2013; Ackerson et al., 2019; Zhang et al., 2020; Atamna et al., 2021; Surie et al., 2023). However, these predictors for severe RSV infections and poor survival may not equate or be the same as the predictors for the hospital LOS. There is a lack of literature assessing the predictors for prolonged hospital LOS among patients hospitalized with RSV infection.

Clinicians must be aware of RSV as a causative agent for severe respiratory infection in older adults despite its lower incidence rates compared to COVID-19 and influenza (Surie et al., 2023). Identifying factors linked to prolonged hospitalization can help with risk stratification for administering FDA-approved RSV vaccines (Papi et al., 2023; Walsh et al., 2023). Therefore, this study aims to identify risk factors associated with prolonged hospital LOS in adult patients admitted with RSV infection.

## Methods

### Study setting and design

This historical cohort study was conducted at five community-based teaching hospitals in Southeast Michigan. Adult patients hospitalized with a laboratory-confirmed diagnosis of RSV between January 1, 2017, and December 31, 2021, were included. Cases with RSV infections were identified using RSV-related International Classification of Diseases, Tenth Revision (ICD-10) codes which included RSV infection (B97.4), RSV pharyngitis (J02.8), RSV bronchitis (J20.5), RSV bronchiolitis (J21.0), RSV laryngotracheobronchitis (J40), RSV pneumonia (J12.1), RSV positive sputum culture (R84.5), viral upper respiratory infection (J06.9), viral pneumonia (J12.9) and viral pharyngitis (J02.9). Then electronic medical records of the patients identified with RSV were reviewed to confirm the laboratory diagnosis of RSV infection. Laboratory testing for RSV utilized a molecular method that involved polymerase chain reaction on a nasal swab sample. The study was reviewed and approved by the Ascension Institutional Review Board (approval number – RMIJ20210469).

### Study subjects

The study subjects included adult patients ages 18 years and older with confirmed RSV who were hospitalized from January 1, 2017, until December 31, 2021. Patients younger than 18 years of age and patients who tested positive but did not require hospitalization were excluded from the study.

### Data collection

The electronic medical records of the hospitalized patients were reviewed for demographic characteristics, clinical characteristics including the comorbidities counted in the Charlson Weighted Index of Comorbidity (CWIC) (Charlson et al., 1987), smoking history, vital signs at the time of presentation as well as within 24 h of hospitalization, laboratory data, radiological images, and length of stay.

### Definitions

“Prolonged LOS” was defined as a hospital stay greater than the mean LOS for the study group. “Viral pneumonia” was defined as an lower respiratory tract infection (LRTI) that had clinical features suggestive of viral infection such as precedence by viral prodrome, lymphopenia, bilateral ground glass opacities or infiltrates seen radiologically that resolved spontaneously without requiring antibiotics. “Fever” was defined as an axillary temperature of 37.5 degrees C or higher. An “abnormal chest x-ray” (CXR) was defined as radiological evidence of new infiltrates or ground glass opacities suggestive of infectious etiology. The presence of atelectasis, pulmonary vascular congestion, and chronic lung changes were not considered as abnormal resulting from infectious etiology. All radiological images were reviewed and compared to previous images if available for the accuracy of the data. The Quick Sequential Organ Failure Assessment (qSOFA) score (0–3) was calculated with the three clinical parameters with one point allotted to each of these parameters: systolic blood pressure ≤ 100 mm Hg, respiratory rate ≥ 22 breaths/min, and altered mental status (Freund et al., 2017). Immunosuppressed status included HIV positivity; neutropenia with absolute neutrophil counts less than 0.50 K/mcL at the time of hospitalization; receipt of steroids (equivalent to at least 15 mg of prednisone per day for at least 14 days consecutively) at the time of hospitalization; receipt of chemotherapy or radiotherapy or anti-tumor necrosis factor-α therapy in the past 3 months; and history of organ transplant.

### Statistical analysis

Descriptive statistics were calculated to characterize the study group. Continuous variables such as age were described as the mean with standard deviation or median with range or interquartile range. Categorical variables were described as frequency distributions. LOS was categorized as below the mean LOS or at the mean or above. Univariable analyses were done using Student’s t-test, analysis of variance followed by multiple pairwise comparisons using the Bonferroni correction of the *p*-value and the chi-squared test. For non-normally distributed data, the Mann–Whitney U test and the Kruskal-Wallis test were used. Multivariable analysis was done using multiple logistic regression. All data were analyzed using SPSS v. 29.0 and a p-value of 0.05 or less was considered to indicate statistical significance.

## Results

**Figure 1:**
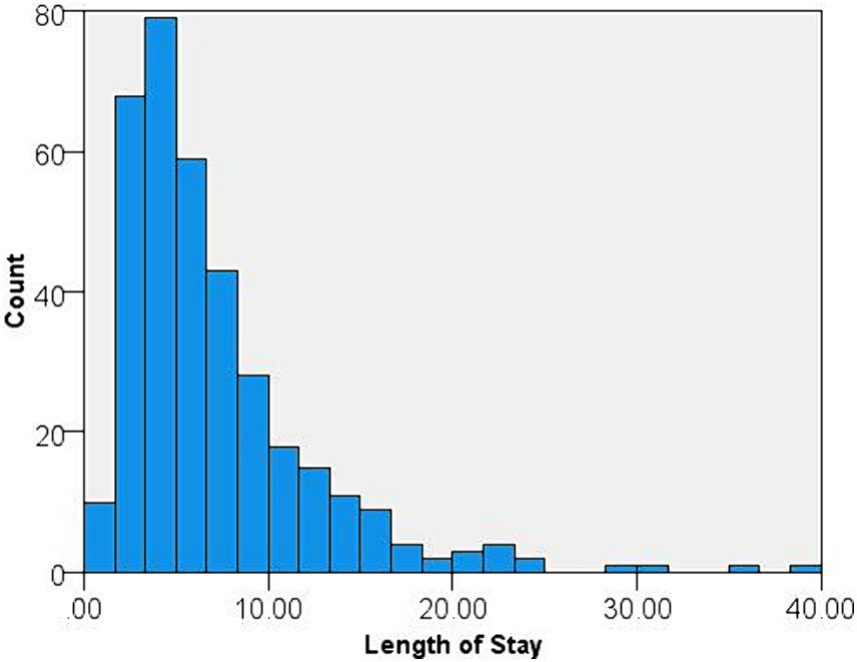
Histogram of the length of stay.

A total of 360 adult patients with confirmed RSV infection were included in the analysis, of which 229 (63.6%) were female and 228 (63.3%) were of white race. The mean age of the study group was 69.9 ± 14.7 years and 239 (66.4%) were 65 years or older. Of these patients, 298 (82.8%) were admitted from home and 48 (13.3%) were residents of nursing homes. A history of smoking (former or current) was present in 202 (56.1%) patients. The mean body mass index (BMI) was 30.6 ± 9.7 kg/m^2^ and the mean Charlson score was 2.2 ± 1.9. Hypertension was the most common comorbidity (66.6%) followed by chronic lung disease (57.4%), diabetes mellitus (30.9%) and CHF (26.2%). The most common symptom was cough (86.9%), followed by shortness of breath (80.1%), nasal congestion (27.5%) and fever (25.4%). The mean hospital LOS was 7.1 ± 5.4 days (Figure 1).

Prolonged LOS was noted in 126 (35.1%) patients. As seen in Table 1, patients with a prolonged LOS were significantly older than those with a LOS of ≤7 days (*p* < 0.001). Patients with prolonged LOS were more likely to have lower mean BMI values (*p* = 0.008) and higher mean CWIC scores (*p* = 0.005). Patients with a history of smoking (*p* = 0.007), myocardial infarction (*p* = 0.043), COPD (*p* = 0.006) and solid organ malignancy (*p* = 0.013) were more likely to have prolonged LOS than patients free from those conditions. Patients who fulfilled the criteria of sepsis on presentation (*p* = 0.032) were more likely to have prolonged LOS. LOS was significantly prolonged among patients who were on home oxygen for chronic lung disease or those who required oxygen at the time of presentation compared to those who did not. Patients with abnormal chest x-rays on presentation and those who received antibiotics at the time of presentation (*p* = 0.006) were more likely to have prolonged LOS.

**Table 1:** Univariable analysis of risk factors at admission associated with prolonged LOS in adult patients with RSV infections.

Characteristics	Total (*n* = 360)	≤ 7 days (*n* = 234)	> 7 days (*n* = 126)	*p* value
Age in years (mean ± SD)	69.9 ± 14.7	67.9 ± 15.9	73.8 ± 11.4	<0.001
Sex				0.49
Male	131 (36.5)	88 (37.8)	43 (34.1)	
Female	228 (63.5)	145 (62.2)	83 (65.9)	
Race				0.85
White	227 (63.2)	146 (62.7)	81 (64.3)	
Black	121 (33.7)	79 (33.9)	42 (33.3)	
Others	11 (3.1)	8 (3.4)	3 (2.4)	
BMI (mean ± SD)	30.6 ± 9.7	31.5 ± 1 0.2	28.9 ± 8.3	0.01
Chronic Comorbid conditions				
Myocardial infarction	24 (6.7)	11 (4.7)	13 (10.3)	0.04
Congestive heart failure	94 (26.2)	58 (24.9)	36 (28.6)	0.45
Asthma	60 (16.7)	48 (20.6)	12 (9.5)	0.007
Chronic obstructive pulmonary disease	145 (40.4)	82 (35.2)	63 (50.0)	0.006
Peripheral arterial disease	21 (5.8)	13 (5.6)	8 (6.3)	0.77
Connective tissue disease	13 (3.6)	10 (4.3)	3 (2.4)	0.36
Diabetes mellitus without complications	60 (16.7)	41 (17.6)	19 (15.1)	0.54
Diabetes mellitus with complications	51 (14.2)	32 (13.7)	19 (15.1)	0.73
Dementia	31 (8.6)	18 (7.7)	13 (10.3)	0.40
Cerebrovascular disease	39 (10.9)	20 (8.6)	19 (15.1)	0.06
Chronic kidney disease	67 (18.7)	44 (18.9)	23 (18.3)	0.88
Human immunodeficiency virus	6 (1.7)	3 (1.3)	3 (2.4)	0.44
Solid malignancy	54 (15)	27 (11.6)	27 (21.4)	0.013
Metastatic disease	10 (2.8)	4 (1.7)	6 (4.8)	0.09
CWIC (mean ± SD)	2.2 ± 1.9	2.03 ± 1.81	2.69 ± 2.20	0.006
Immunosuppressed status	41 (11.4)	26 (11.2)	15 (11.9)	0.83
Hemodialysis	16 (4.5)	10 (4.3)	6 (4.8)	0.84
Smoking	208 (57.9)	121 (51.9)	87 (69)	0.002
Patients on home oxygen	64 (17.8)	32 (13.7)	32 (25.4)	0.006
Systolic BP on admission <100 mm Hg	12 (3.3)	5 (2.1)	7 (5.6)	0.09
RR on admission >22 breaths/min	165 (46)	102 (43.8)	63 (50.0)	0.26
Oxygen requirement at presentation	210 (58.5)	124 (53.2)	86 (68.3)	0.006
qSOFA	19 (5.3)	8 (3.4)	11 (8.7)	0.03
Abnormal chest x-ray on admission	133 (37.5)	76 (33.2)	57 (45.3)	0.09

n, Number, SD, Standard deviation, BMI, Body mass index, CWIC, Charlson weighted index of comorbidity, BP, Blood pressure, RR, Respiratory rate, qSOFA, Quick sepsis related organ failure assessment.

From multivariable logistic regression using a forward likelihood ratio algorithm, the independent predictors for prolonged hospital LOS were age at admission (OR = 1.04, *p* < 0.001), current or former smoker (OR = 2.22, *p* = 0.001), CWIC score (OR = 1.13, *p* = 0.036) and abnormal CXR (OR = 1.62, *p* = 0.043) (Table 2). Every one unit increase in age and CWIC increased the risk for prolonged hospital LOS by 4.0 and 13.2%, respectively. A history of current or former smoking increased the risk 2.2 times and an abnormal CXR on admission increased the risk 62%.

**Table 2:** Multivariable analysis of risk factors at admission associated with Prolonged LOS in adult patients with RSV infection.

Variable	Odds Ratio (95% CI)	*p* value
Age at admission	1.04 (1.02–1.05)	<0.001
Smoking	2.22 (1.36–3.63)	0.001
CWIC score	1.13 (1.01–1.27)	0.036
Abnormal CXR	1.62 (1.02–2.57)	0.043

CI, Confidence interval, CWIC, Charlson weighted index of comorbidity, CXR, Chest x-ray.

## Discussion

Our study found that older age, history of smoking, a more significant burden of comorbidities, and the presence of radiological evidence of pneumonia at the time of presentation were independent predictors for prolonged LOS in adult patients hospitalized because of RSV infections. According to studies conducted in the last decades, the mean LOS for adults with RSV infection is 4–21 days (Lee et al., 2013; Volling et al., 2014; Loubet et al., 2017; Yoon et al., 2020; Hartnett et al., 2022; Walsh et al., 2022). The mean LOS in our study was 7.1 ± 5.4 days, which is relatively shorter than that of other studies (Lee et al., 2013; Loubet et al., 2017; Yoon et al., 2020).

In our study, age on admission was an independent predictor for prolonged LOS. Every one-unit increase in age increases the risk for prolonged hospital LOS by 4.0%. It has been observed that patients who were hospitalized for RSV infection were significantly older than those who were outpatients. This underscores the significance of detecting RSV early and treating it promptly, particularly in those at higher risk due to age-related decline in immunity. Timely intervention can help prevent hospitalization, reduce healthcare costs, and, most importantly, save lives (Walsh et al., 2013). Higher median hospital LOS has been reported among patients with comorbid lung diseases, immunocompromised states, and older adults compared to other adult patients (Walsh et al., 2022). Belongia et al. noted serious outcomes in terms of hospitalizations and pneumonia more frequently in patients >75 years of age (Belongia et al., 2018). RSV infection was associated with longer hospital stays (≥ 7 days), pneumonia, ICU admission, COPD exacerbation, and higher mortality within one year of admission compared to influenza in hospitalized adults aged 60 years or older (Ackerson et al., 2019). This may be a result of weaker immune responses as aging has been linked to declining immune competence (Goronzy et al., 2015). In May 2023, the Food and Drug Administration approved two RSV vaccines for the prevention of LRTI caused by RSV in individuals 60 years and older (Papi et al., 2023; Walsh et al., 2023). In June 2023, the Centers for Disease Control and Prevention recommended a single dose of RSV vaccine for adults ≥60 years old using shared clinical decision-making between patients and clinicians (Melgar et al., 2023). These vaccines can potentially change the landscape with possible reductions in the number of hospitalizations for RSV infections.

The CWIC score is a validated method to predict mortality by measuring comorbid conditions. It is widely used in research to account for the burden of multiple medical conditions (Charlson et al., 1987). Our study found that patients with several medical conditions were more likely to experience extended hospital stays, as indicated by higher CWIC scores. Patients with myocardial infarction, COPD, and solid malignancy were more likely to have extended hospital stays. Epidemiological evidence suggests that people with underlying chronic conditions are at increased risk for severe disease, prolonged hospital stay and death from RSV infection (Belongia et al., 2018; Yoon et al., 2020). A study on Korean adults revealed that solid cancer and hematological malignancy were risk factors for RSV pneumonia and related to poor outcomes of RSV infection (Yoon et al., 2020). Adults with CHF had a higher rate of RSV-associated hospitalization than those without CHF (Branche et al., 2022; Kujawski et al., 2022). For adults ages 65 years and above, the rate of RSV hospitalization is 3.5 times higher for those with CHF, while for adults below 65, the RSV rate is 14.3 times higher for those with CHF (Kujawski et al., 2022). RSV is known to be associated with acute exacerbations of COPD (Seemungal et al., 2001; Zwaans et al., 2014). During acute exacerbation of COPD patients requiring hospitalization, RSV is reportedly the most common non-influenza respiratory viral infection (Mulpuru et al., 2022). Persistent RSV detection in stable COPD patients has been shown to cause airway inflammation as measured by sputum interleukin (IL)-6, IL-8, and myeloperoxidase levels and a rapid decline in lung functions, particularly forced expiratory volume in one second (FEV1) (Seemungal et al., 2001). This chronic infective state is thought to occur because of a reduction in the production of interferons (β and λ). This interferonopathy can trigger cell senescence and autoimmune-mediated lung damage, leading to poor pulmonary outcomes (Ruggeri and Caramori, 2019). Patients hospitalized with RSV are at increased risk for requiring oxygen support, non-invasive ventilation and ICU admission compared to those hospitalized with COVID-19 or influenza (Surie et al., 2023).

In our study, patients with a history of current or former smoking were at increased risk of prolonged LOS. Because of the study’s retrospective nature, it was hard to quantify the tobacco exposure. Cigarette smoking is associated with an increased risk and severity of viral respiratory infections such as RSV (Cohen et al., 1993). Smoking triggers necrotic cell death rather than apoptotic cell death, which is associated with increased viral replication (Groskreutz et al., 2009). This was reflected by the findings of increased viral titers in airway epithelial cells following exposure to cigarette smoke extract. Apoptosis is a more efficient and less inflammatory way of clearing virally infected cells from the airway compared to necrosis. Smoking also increases the risk for asthma among young adults who had RSV LRTIs in early life compared to those who did not have RSV-LRTIs (Voraphani et al., 2014).

Our study found that people who had new CXR findings in terms of infiltrates, consolidation, or ground glass opacities were more likely to have prolonged hospital LOS. Earlier studies have reported about 40–60% of hospitalized patients with RSV-related illnesses have clinical evidence for pneumonia (Volling et al., 2014; Yoon et al., 2020). Multiple studies have shown that RSV infections are associated with a higher incidence of hospitalization risks, pneumonia occurrences, and higher prescriptions for antibiotics than influenza infections (Lee et al., 2013; Ackerson et al., 2019; Zhang et al., 2020; Atamna et al., 2021; Hämäläinen et al., 2022; Surie et al., 2023). RSV’s progression from the upper respiratory tract to the lower airways initiates a series of events. It first targets ciliated and alveolar epithelial type II cells, triggering the release of cytokines and chemokines (Manti and Piedimonte, 2022). It leads to an influx of inflammatory cells into the infected lung tissue, followed by epithelial cell shedding. This process accelerates the clearance of virus-infected cells from the airway mucosa and contributes to acute obstruction of the distal airways, ultimately leading to severe reactive airway disease (Liesman et al., 2014; Manti and Piedimonte, 2022). Patients with RSV who require mechanical ventilation are also at increased risk of complications, including bronchospasm, lung atelectasis, and ventilator-associated pneumonia (Santus et al., 2023).

The strength of our study includes large sample size, multicenter study design, and data collection over 5 years. The data were collected and interpreted by a single person, eliminating errors with multiple data collectors. The main limitation of our study was that we collected data retrospectively. Despite this limitation, we made every effort to ensure the accuracy and reliability of our findings. Our study still provides valuable insights and highlights the need for further research. Second, the study included patients from the five hospitals in Southeast Michigan, so the findings may not be generalizable. The study only included hospitalized patients, so the findings cannot be generalized to the whole population as most RSV patients do not require hospitalization.

In conclusion, our study found that age on admission, positive smoking history, higher CWIC score, and presence of abnormal chest imaging suggestive of pneumonia on presentation were significantly associated with prolonged hospital LOS among adult patients with RSV infection. These findings highlight the significance of promptly recognizing and implementing effective interventions to mitigate the duration of hospitalization for adult patients suffering from RSV infection. Swift action and early measures can significantly impact the patient’s recovery process, reducing the burden on healthcare systems and improving the quality of care provided.

## Data availability statement

The original contributions presented in the study are included in the article/supplementary material, further inquiries can be directed to the corresponding author.

## Ethics statement

The studies involving humans were approved by Ascension Institutional Review Board. The studies were conducted in accordance with the local legislation and institutional requirements. The ethics committee/institutional review board waived the requirement of written informed consent for participation from the participants or the participants’ legal guardians/next of kin because the research involves no more than minimal risk to the subjects.

## Author contributions

AM: Data curation, Investigation, Writing – original draft. SS: Formal analysis, Methodology, Validation, Writing – review & editing. MS: Supervision, Writing – review & editing. LJ: Supervision, Writing – review & editing. LS: Project administration, Supervision, Writing – review & editing. AB: Conceptualization, Methodology, Project administration, Supervision, Writing – review & editing.

## References

[ref1] Ackerson B. Tseng H. F. Sy L. S. Solano Z. Slezak J. Luo Y.. (2019). Severe morbidity and mortality associated with respiratory syncytial virus versus influenza infection in hospitalized older adults. Clin. Infect. Dis. 69, 197–203. doi: 10.1093/cid/ciy991, PMID: 30452608 PMC6603263

[ref2] Atamna A. Babich T. Froimovici D. Yahav D. Sorek N. Ben-Zvi H.. (2021). Morbidity and mortality of respiratory syncytial virus infection in hospitalized adults: comparison with seasonal influenza. Int. J. Infect. Dis. 103, 489–493. doi: 10.1016/j.ijid.2020.11.185, PMID: 33249288

[ref3] Belongia E. A. King J. P. Kieke B. A. Pluta J. Al-Hilli A. Meece J. K.. (2018). Clinical features, severity, and incidence of RSV illness during 12 consecutive seasons in a community cohort of adults ≥60 years old. Open forum. Infect. Dis. 5:ofy316. doi: 10.1093/ofid/ofy316, PMID: 30619907 PMC6306566

[ref4] Branche A. R. Saiman L. Walsh E. E. Falsey A. R. Sieling W. D. Greendyke W.. (2022). Incidence of respiratory syncytial virus infection among hospitalized adults, 2017-2020. Clin. Infect. Dis. 74, 1004–1011. doi: 10.1093/cid/ciab595 34244735

[ref5] Centers for Disease Control and Prevention. Respiratory syncytial virus (RSV) surveillance and research. Available at: https://www.cdc.gov/rsv/research/index.html. (Accessed December 8, 2023).

[ref6] Charlson M. E. Pompei P. Ales K. L. MacKenzie C. R. (1987). A new method of classifying prognostic comorbidity in longitudinal studies: development and validation. J. Chronic Dis. 40, 373–383. doi: 10.1016/0021-9681(87)90171-8, PMID: 3558716

[ref7] Chatzis O. Darbre S. Pasquier J. Meylan P. Manuel O. Aubert J. D.. (2018). Burden of severe RSV disease among immunocompromised children and adults: a 10 year retrospective study. BMC Infect. Dis. 18:111. doi: 10.1186/s12879-018-3002-3, PMID: 29510663 PMC5838875

[ref8] Choi Y. Hill-Ricciuti A. Branche A. R. Sieling W. D. Saiman L. Walsh E. E.. (2022). Cost determinants among adults hospitalized with respiratory syncytial virus in the United States, 2017-2019. Influenza Other Respir. Viruses 16, 151–158. doi: 10.1111/irv.12912, PMID: 34605182 PMC8692803

[ref9] Cohen S. Tyrrell D. A. Russell M. A. Jarvis M. J. Smith A. P. (1993). Smoking, alcohol consumption, and susceptibility to the common cold. Am. J. Public Health 83, 1277–1283. doi: 10.2105/ajph.83.9.1277, PMID: 8363004 PMC1694990

[ref10] Englert J. Davis K. M. Koch K. E. (2001). Using clinical practice analysis to improve care. Jt. Comm. J. Qual. Improv. 27, 291–301. doi: 10.1016/s1070-3241(01)27025-4 11402776

[ref11] Freund Y. Lemachatti N. Krastinova E. Van Laer M. Claessens Y. E. Avondo A.. (2017). Prognostic accuracy of Sepsis-3 criteria for in-hospital mortality among patients with suspected infection presenting to the emergency department. JAMA 317, 301–308. doi: 10.1001/jama.2016.20329, PMID: 28114554

[ref12] Goronzy J. J. Fang F. Cavanagh M. M. Qi Q. Weyand C. M. (2015). Naive T cell maintenance and function in human aging. J. Immunol. 194, 4073–4080. doi: 10.4049/jimmunol.1500046, PMID: 25888703 PMC4452284

[ref13] Groskreutz D. J. Monick M. M. Babor E. C. Nyunoya T. Varga S. M. Look D. C.. (2009). Cigarette smoke alters respiratory syncytial virus-induced apoptosis and replication. Am. J. Respir. Cell Mol. Biol. 41, 189–198. doi: 10.1165/rcmb.2008-0131OC, PMID: 19131644 PMC2715908

[ref14] Hämäläinen A. Savinainen E. Hämäläinen S. Sivenius K. Kauppinen J. Koivula I.. (2022). Disease burden caused by respiratory syncytial virus compared with influenza among adults: a retrospective cohort study from eastern Finland in 2017-2018. BMJ Open 12:e060805. doi: 10.1136/bmjopen-2022-060805, PMID: 36535718 PMC9764619

[ref15] Hartnett J. Donga P. Ispas G. Vandendijck Y. Anderson D. House S.. (2022). Risk factors and medical resource utilization in US adults hospitalized with influenza or respiratory syncytial virus in the hospitalized acute respiratory tract infection study. Influenza Other Respir. Viruses 16, 906–915. doi: 10.1111/irv.12994, PMID: 35474419 PMC9343339

[ref16] Kieke B. A. Belongia E. A. McClure D. L. Shinde V. (2020). Prediction of serious RSV-related outcomes in older adults with outpatient RSV respiratory illness during 12 consecutive seasons. Influenza Other Respir. Viruses 14, 479–482. doi: 10.1111/irv.12751, PMID: 32390298 PMC7431638

[ref17] Kujawski S. A. Whitaker M. Ritchey M. D. Reingold A. L. Chai S. J. Anderson E. J.. (2022). Rates of respiratory syncytial virus (RSV)-associated hospitalization among adults with congestive heart failure-United States, 2015-2017. PLoS One 17:e0264890. doi: 10.1371/journal.pone.0264890, PMID: 35263382 PMC8906631

[ref18] Lee N. Lui G. C. Wong K. T. Li T. C. Tse E. C. Chan J. Y.. (2013). High morbidity and mortality in adults hospitalized for respiratory syncytial virus infections. Clin. Infect. Dis. 57, 1069–1077. doi: 10.1093/cid/cit471 23876395

[ref19] Liesman R. M. Buchholz U. J. Luongo C. L. Yang L. Proia A. D. DeVincenzo J. P.. (2014). RSV-encoded NS2 promotes epithelial cell shedding and distal airway obstruction. J. Clin. Invest. 124, 2219–2233. doi: 10.1172/JCI72948, PMID: 24713657 PMC4001550

[ref20] Loubet P. Lenzi N. Valette M. Foulongne V. Krivine A. Houhou N.. (2017). FLUVAC study group. Clinical characteristics and outcome of respiratory syncytial virus infection among adults hospitalized with influenza-like illness in France. Clin. Microbiol. Infect. 23, 253–259. doi: 10.1016/j.cmi.2016.11.014, PMID: 27903461 PMC7128342

[ref21] Manti S. Piedimonte G. (2022). An overview on the RSV-mediated mechanisms in the onset of non-allergic asthma. Front. Pediatr. 10:998296. doi: 10.3389/fped.2022.998296, PMID: 36204661 PMC9530042

[ref22] Melgar M. Britton A. Roper L. E. Talbot H. K. Long S. S. Kotton C. N.. (2023). Use of respiratory syncytial virus vaccines in older adults: recommendations of the advisory committee on immunization practices - United States, 2023. MMWR Morb. Mortal Wkly. Rep. 72, 793–801. doi: 10.15585/mmwr.mm7229a4, PMID: 37471262 PMC10360650

[ref23] Mulpuru S. Andrew M. K. Ye L. Hatchette T. LeBlanc J. El-Sherif M.. (2022). Serious outcomes surveillance and Canadian immunization research network (CIRN) investigators. Impact of respiratory viral infections on mortality and critical illness among hospitalized patients with chronic obstructive pulmonary disease. Influenza Other Respir. Viruses 16, 1172–1182. doi: 10.1111/irv.13050, PMID: 36069141 PMC9530520

[ref24] Njue A. Nuabor W. Lyall M. Margulis A. Mauskopf J. Curcio D.. (2023). Systematic literature review of risk factors for poor outcomes among adults with respiratory syncytial virus infection in high-income countries. Open forum. Infect. Dis. 10:ofad513. doi: 10.1093/ofid/ofad513, PMID: 38033988 PMC10686344

[ref25] Papi A. Ison M. G. Langley J. M. Lee D. G. Leroux-Roels I. Martinon-Torres F.. (2023). Respiratory syncytial virus Prefusion F protein vaccine in older adults. N. Engl. J. Med. 388, 595–608. doi: 10.1056/NEJMoa2209604 36791160

[ref26] Ruggeri P. Caramori G. (2019). Interferonopathy: a potential link between innate immunity and autoimmunity in the pathogenesis of COPD. Am. J. Physiol. Lung Cell. Mol. Physiol. 317, L888–L890. doi: 10.1152/ajplung.00439.2019, PMID: 31664861

[ref27] Sah R. Zaman K. Mohanty A. al-Ahdal T. Awad H. Padhi B. K.. (2023). Respiratory syncytial virus with ongoing COVID-19: is it an emerging threat? Ann. Med. Surg. 85, 67–70. doi: 10.1097/MS9.0000000000000153, PMID: 36742116 PMC9893426

[ref28] Santus P. Radovanovic D. Gismondo M. R. Rimoldi S. G. Lombardi A. Danzo F.. (2023). Respiratory syncytial virus burden and risk factors for severe disease in patients presenting to the emergency department with flu-like symptoms or acute respiratory failure. Respir. Med. 218:107404. doi: 10.1016/j.rmed.2023.107404 37683776

[ref29] Seemungal T. A. R. Harper-Owen R. Bhowmik A. Moric I. Sanderson G. Message S.. (2001). Respiratory viruses, symptoms, and inflammatory markers in acute exacerbations and stable chronic obstructive pulmonary disease. Am. J. Respir. Crit. Care Med. 164, 1618–1623. doi: 10.1164/ajrccm.164.9.2105011, PMID: 11719299

[ref30] Surie D. Yuengling K. A. DeCuir J. Zhu Y. Gaglani M. Ginde A. A.. (2023). Disease severity of respiratory syncytial virus compared with COVID-19 and influenza among hospitalized adults aged ≥60 years - IVY network, 20 U.S. states, February 2022-may 2023. MMWR Morb. Mortal Wkly. Rep. 72, 1083–1088. doi: 10.15585/mmwr.mm7240a2 37796753 PMC10564326

[ref31] Volling C. Hassan K. Mazzulli T. Green K. al-den A. Hunter P.. (2014). Respiratory syncytial virus infection-associated hospitalization in adults: a retrospective cohort study. BMC Infect. Dis. 14:665. doi: 10.1186/s12879-014-0665-2, PMID: 25494918 PMC4269936

[ref32] Voraphani N. Stern D. A. Wright A. L. Guerra S. Morgan W. J. Martinez F. D. (2014). Risk of current asthma among adult smokers with respiratory syncytial virus illnesses in early life. Am. J. Respir. Crit. Care Med. 190, 392–398. doi: 10.1164/rccm.201311-2095OC, PMID: 24927374 PMC4214125

[ref33] Walsh E. Lee N. Sander I. Stolper R. Zakar J. Wyffels V.. (2022). RSV-associated hospitalization in adults in the USA: a retrospective chart review investigating burden, management strategies, and outcomes. Health Sci. Rep. 5:e556. doi: 10.1002/hsr2.556, PMID: 35509398 PMC9059216

[ref34] Walsh E. E. Pérez Marc G. Zareba A. M. Falsey A. R. Jiang Q. Patton M.. (2023). Efficacy and safety of a bivalent RSV Prefusion F vaccine in older adults. N. Engl. J. Med. 388, 1465–1477. doi: 10.1056/NEJMoa2213836, PMID: 37018468

[ref35] Walsh E. E. Peterson D. R. Kalkanoglu A. E. Lee F. E. Falsey A. R. (2013). Viral shedding, and immune responses to respiratory syncytial virus infection in older adults. J. Infect. Dis. 207, 1424–1432. doi: 10.1093/infdis/jit038, PMID: 23382572 PMC3610422

[ref36] Yoon J. G. Noh J. Y. Choi W. S. Park J. J. Suh Y. B. Song J. Y.. (2020). Clinical characteristics and disease burden of respiratory syncytial virus infection among hospitalized adults. Sci. Rep. 10:12106. doi: 10.1038/s41598-020-69017-8, PMID: 32694533 PMC7374583

[ref37] Zhang Y. Wang Y. Zhao J. Xiong Z. Fan Y. Zhang W.. (2020). Severity and mortality of respiratory syncytial virus vs influenza a infection in hospitalized adults in China. Influenza Other Respir. Viruses 14, 483–490. doi: 10.1111/irv.12754, PMID: 32449300 PMC7431648

[ref38] Zwaans W. A. Mallia P. van Winden M. E. Rohde G. G. (2014). The relevance of respiratory viral infections in the exacerbations of chronic obstructive pulmonary disease-a systematic review. J. Clin. Virol. 61, 181–188. doi: 10.1016/j.jcv.2014.06.025, PMID: 25066886 PMC7106508

